# Endoscopic third ventriculostomy for management of vein of Galen aneurysmal malformation-associated hydrocephalus with spontaneous thrombosis: case report

**DOI:** 10.3389/fped.2026.1974046

**Published:** 2026-10-07

**Authors:** Arhum Naeem, Kelsi Chesney, Sebastian Salas-Vega, Dallas Soyland, Saige A. Teti, Sonora Q. Robles, Brannan ONeill, Daniel Donoho, Hasan R. Syed, Chima O. Oluigbo, John S. Myseros, Robert F. Keating, Kyle Spagnolo, Jonathan Murnick, Monica Pearl, Abdullah H. Feroze

**Affiliations:** Department of Neurosurgery, Children's National Hospital, Washington, DC, United States

**Keywords:** cerebrovascular malformation, endovascular embolization, hydrocephalus, pediatric neurosurgery, vein of galen aneurysmal malformation, ventriculoperitoneal (VP) shunt

## Abstract

Vein of Galen aneurysmal malformations (VGAMs) are rare cerebrovascular anomalies due to lack of involution of the prosencephalic vein of Markowski in early gestation. Children born with such pathology may present with high-output cardiac failure, developmental delay, seizures, and hydrocephalus (Lasjaunias et al., 2006; Berenstein et al., 2010). Hydrocephalus may present due to aqueductal obstruction, intraventricular hemorrhage, or venous hypertension leading to absorptive deficit. Historical data suggests extremely high rates of complications associated with ventriculoperitoneal shunting when required in VGAM (Jea et al., 2010). Herein, we describe our experience with endoscopic third ventriculostomy for treatment of a partially embolized VGAM patient presenting with acute decline secondary to headaches, vomiting, and papilledema with evidence of symptomatic and radiographic resolution at two-month followup. Six-month MRI/MRA followup confirms persistent obliteration of the cerebrovascular lesion.

## Background

Vein of Galen malformations are challenging fistulous lesions of early childhood due to a failure of involution of the prosencephalic vein of Markowski by the third month of gestation. Such lesions can manifest with hydrocephalus due to derangements in hydrodynamics secondary to venous hypertension. Due to the high-flow fistulous nature of such cerebrovascular lesions, venous hypertension secondarily disturbs cerebrospinal fluid (CSF) absorption and manifests in ventriculomegaly and, rarely, symptomatic hydrocephalus (1–3). Of note, obstructive hydrocephalus due to compression of the aqueduct of Sylvius due to large VOGM lesions or embolic materials may also serve as an alternative etiology of hydrocephalus, but this is far less common than instances of hydrodynamic imbalance due to venous hypertension. In the setting of inadequate venous outflow to match arteriovenous shunting, intracranial hypertension and hydrocephalus may manifest as seizures, hemorrhage, or focal neurologic deficits. Chronic venous congestion may induce calcification of the subcortical white matter, often found in the venous watershed regions of the brain between the superficial and deep venous systems, resulting in developmental delay. First line treatment to avoid such pathology includes transarterial embolization of VGAM inflow in effort to restore the hydrodynamic balance as able (4).

Historically, in cases where restoration of the hydrodynamic balance was not possible and symptomatic hydrocephalus required treatment, ventriculoperitoneal shunting was standard of care. However, multiple studies describe the high rate of complications with shunting in VOGM patients, with rates of complication as high as 70% including subdural hematoma/hygroma, intraventricular hemorrhage, and status epilepticus (5). Recent literature has highlighted an important trend in the management of pediatric vein of Galen patients, where an increasing body of work suggests that endoscopic third ventriculostomy (ETV) should be considered first-line treatment for management of symptomatic hydrocephalus in this patient subpopulation when endovascular embolization is considered futile or too high risk (6–8). The risks of ventriculoperitoneal shunting in this subpopulation have been known for decades, and as both neuroendoscopic and interventional neuroradiology equipment and technique have enjoyed many advances over the past few years, there is an increasing argument for clinical equipoise in this space. Our case herein demonstrates not only ETV to be an appropriate treatment for hydrocephalus in this setting but also given coincidental thrombosis of the VGAM at two-month radiographic followup suggests that equilibration of pressures across the endoscopic ostomy at the tuber cinereum might potentially promote thrombosis of such lesions, particularly in the case of prior partial embolization as herein (Figure 3).

## Case presentation

A 5-year-old boy with a choroidal-type vein of Galen malformation and three prior transarterial embolizations (Figure 1) presented with several days of headaches and vomiting. His last transarterial embolization of the VOGM had been eight months prior to presentation to the emergency department.

**Figure 1:**
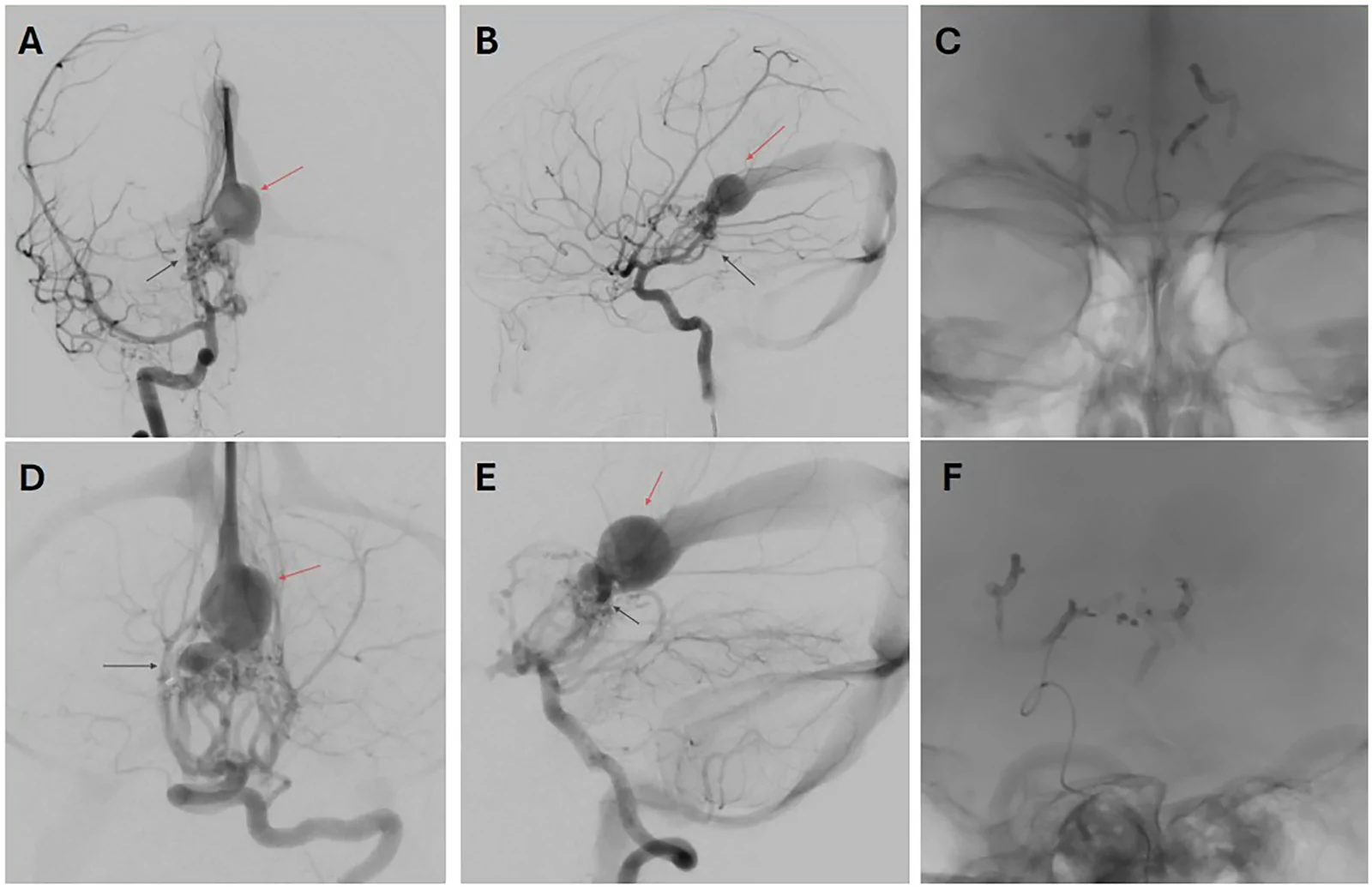
Representative imaging of choroidal type vein of galen malformation (VGAM) with drainage into dilated prosencephalic vein of markowski (red arrow) via multiple feeders from anterior choroidal, posterior choroidal, and transmesencephalic feeding branches (black arrow) upon right internal carotid artery injection **(A,B)** and left vertebral artery injection **(D,E)** with evidence of prior nBCA embolization glue casts **(C,F)**.

On ophthalmologic examination, there was evidence of papilledema and disk edema. MR imaging of the brain demonstrated known severe ventriculomegaly but with transependymal flow concerning for acute hydrocephalus (Figure 2).

**Figure 2:**
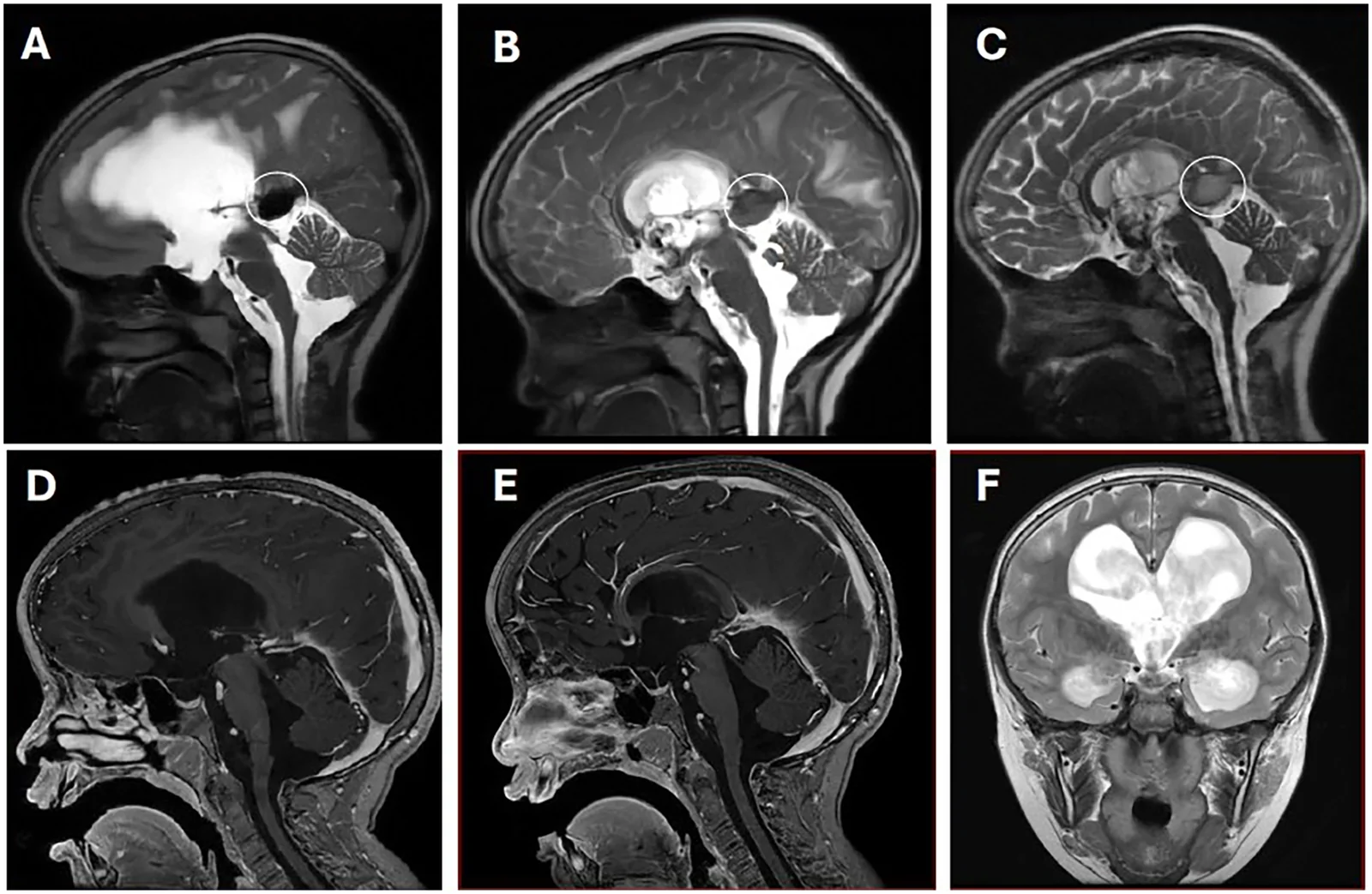
5-year-old child presenting with an acute exacerbation of chronic ventriculomegaly with periventricular edema treated with endoscopic third ventriculostomy (ETV). Sagittal T2-weighted **(A–C)** and sagittal T1-post contrast **(D)** MRIs are suggestive of progressive thrombosis of the VGAM following ETV (circled). A preoperative sagittal T2 demonstrates robust flow void in the VGAM with periventricular edema **(A)** T2 hyperintensity increases in the VGAM on post-operative day (POD) 1 **(B)** through POD 26 **(C)**, resulting ultimately in thrombosis/regression of the cerebrovascular lesion by two months postoperatively **(D)** Lesion remains obliterated on six-month postoperative imaging **(E)** with evidence of persistent flow artifact across membrane of Liliequist on coronal T2 view suggestive of patent ostomy at ETV site **(F).**

**Figure 3:**
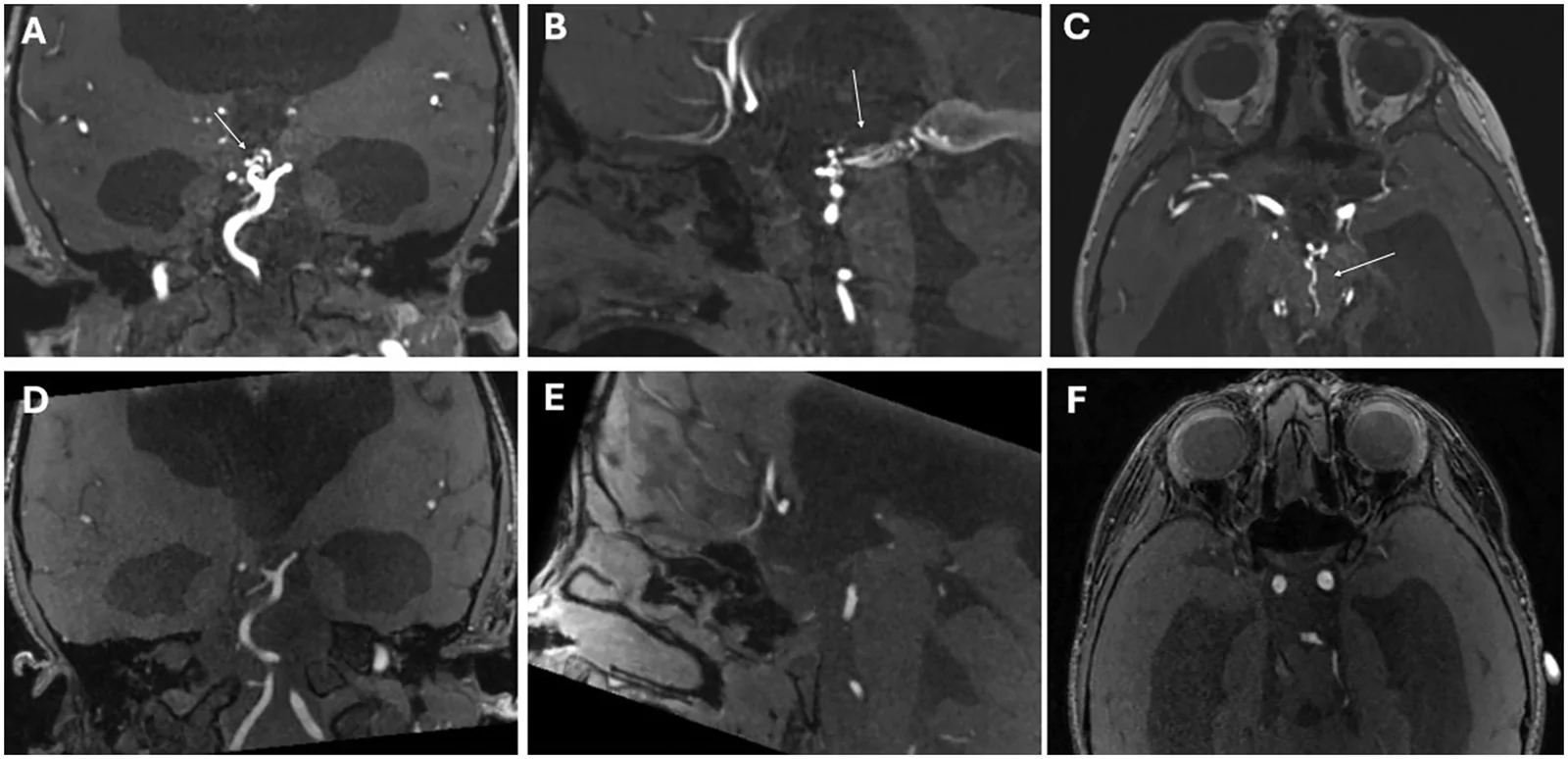
MR angiography of the same patient comparing preoperative imaging **(A–C)** versus postoperative imaging **(D–F)** following ETV. Postoperatively, spontaneous thrombosis manifests as loss of flow-related enhancement throughout numerous tortuous inflow arterial vessels from the interpeduncular, mesencephalic, and quadrigeminal cisterns (arrows) as well as within the venous sac itself.

In the setting of acute decompensation with symptomatic hydrocephalus with headaches, nausea, vomiting, papilledema, and new periventricular edema in the setting of baseline ventriculomegaly, endoscopic third ventriculostomy (ETV) was proposed as the preferred option over ventriculoperitoneal shunting. Risks, benefits, and alternatives to both surgical options were reviewed with the patient's family, and they asked us to proceed forward with ETV. Despite risks associated with neuroendoscopy in the setting of aberrant angioarchitecture in VGAM patients, we felt the risks associated with ventriculoperitoneal shunting in this patient population was far greater as aforementioned. ETV success score was estimated to be 70.

## Outcome and followup

ETV procedure was performed with use of a rigid Lotta endoscope (Karl Storz, Germany) by right frontal trajectory in standard fashion using a 12.5F introducer sheath. The procedure was tolerated without complication, and the patient discharged home on postoperative day one with improvement of headaches, nausea, and vomiting. At two-week followup, clinical symptoms of hydrocephalus had completely resolved with no further headaches, nausea, or vomiting.

At two-month MRI/MRA followup, there was incidental evidence of complete thrombosis of the venous sac and non-opacification of arterial feeders to the malformation, suggestive of interval thrombosis of the lesion, posited to be potentially due to equilibration of intracranial pressures across the membrane of Liliequist following ETV promoting subsequent thrombosis. At last followup six months postoperatively, the patient remains well without headaches, nausea, vomiting, or other symptoms and repeat MRI brain demonstrates no filling of the vein of Galen malformation. MRA brain demonstrates confirmed persistent loss of flow-related enhancement throughout numerous tortuous inflow arterial vessels from the interpeduncular, mesencephalic, and quadrigeminal cisterns as well as within the venous sac itself as also seen on two-month postoperative imaging. In addition, T2 flow artifact suggests persistent patency of the third ventriculostomy site (Figures 2, 3).

## Discussion

Recent reports describe the safety of endoscopic third ventriculostomy in the management of VOGM-associated hydrocephalus. Review of literature demonstrates nine publications with a total of twenty pediatric patients who underwent successful with ETV for VGAM-associated hydrocephalus (Supplementary STable 1) ranging from ages of 3.5 months to teenage years (8–16). Four procedural-related complications were described across these twenty patients, including intraprocedural venous injury, truncal ataxia, seizures, and one episode of delayed ETV failure requiring ultimate placement of ventriculoperitoneal shunt (Supplementary Table S1). Our report herein is the second to describe spontaneous thrombosis of the VGAM in delayed fashion following prior endovascular embolization and subsequent ETV for hydrocephalus treatment as previously reported by Guil-Ibanez *et al*. in the care of 5-month old child with a mural type VGAM. Our report herein differs due to delayed presentation of a choroidal type VGAM followed staged transarterial embolization.

As aforementioned, the most optimal management strategy to reduce venous hypertension secondary to VGAM pathophysiology is by early endovascular embolization. If endovascular treatment or diagnosis is delayed until following symptomatic hydrocephalus, salvage therapies such as ventriculoperitoneal shunting or ETV are the most appropriate options. We posit that the spontaneous thrombosis of the lesion observed herein is potentially due to equilibration of pressures above and below the tuber cinereum caused by ETV with impact on degree of local arteriovenous shunting and subsequent lesional thrombosis. As Figure 2 demonstrates, there is clear yet gradual change in T2 signal intensity of the VOGM lesion across several weeks, from imaging from postoperative day 1 (2B) and day 26 (2C) compared to preoperative imaging, which suggests changes in lesional hemodynamics at that time. Furthermore, intraoperative endoscopy demonstrated persistent thalamoperforators feeding the galenic complex under direct visualization were critical to maneuver around in the completion of the ETV (Figure 4). Yet, ultimately, the potential for spontaneous thrombosis prior to ETV cannot be fully excluded.

**Figure 4:**
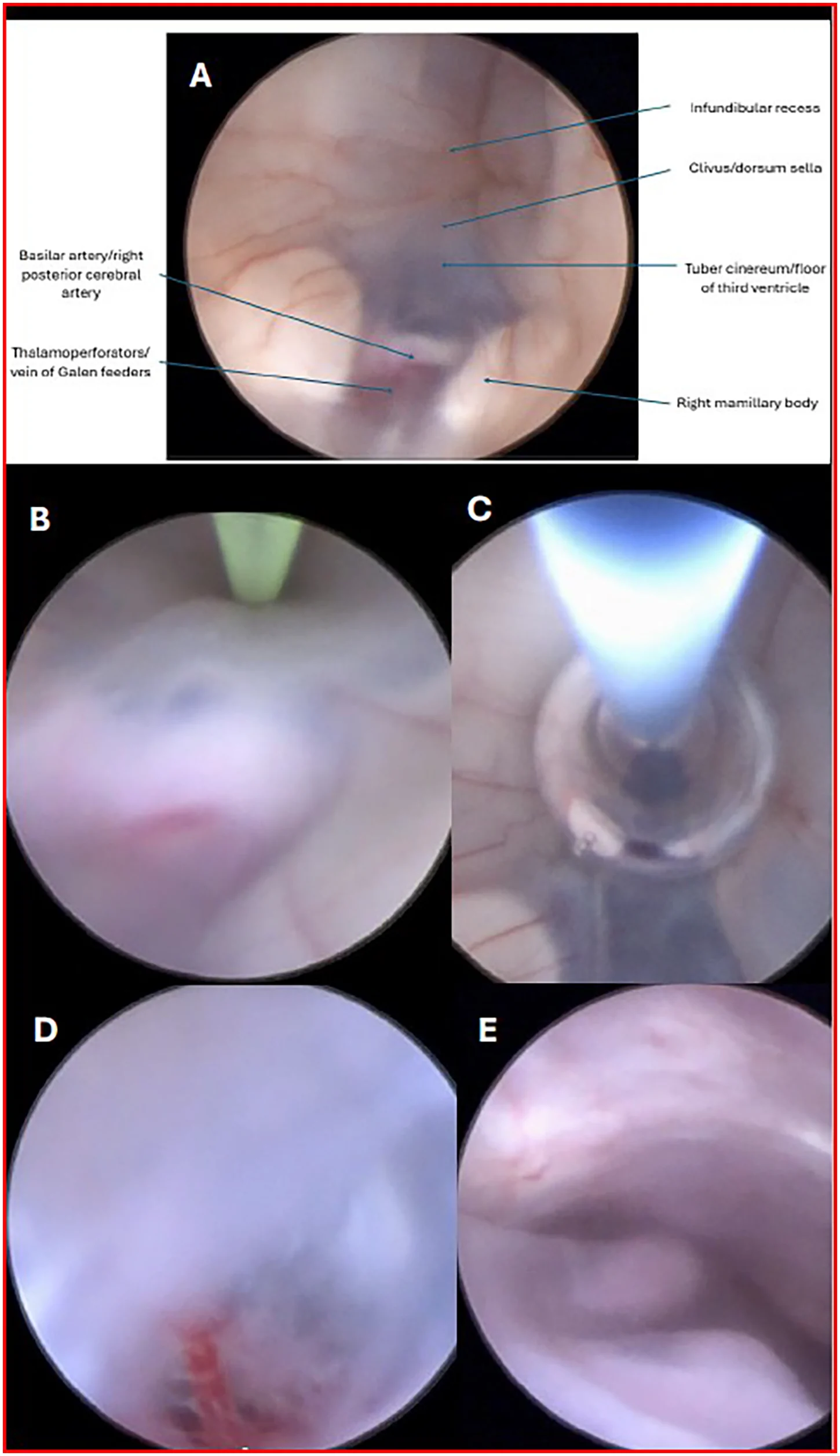
Intraoperative view during endoscopic third ventriculostomy demonstrating critical neurovascular structures in choroidal type vein of Galen aberrant architecture (**A**), including ostomy at the tuber cinereum (**B, C**), thalamoperforators feeding the known malformation (**D**), and final view in the prepontine space following successful ostomy (**E**).

Some clinicians advocate for choroid plexus cauterization (CPC) as part of any ETV procedure in attempt to improve long-term results. As widely recognized, CPC requires an experienced operator. It does so even furthermore in an instance of aberrant angioarchitecture at the tuber cinereum (Figure 4) and surrounding regions providing arterial inflow to the vein of Galen malformation in a setting of elevated arterial pressure compounded by known venous hypertension. Inadvertent injury to such vessels with endoscopy or with attempted coagulation and avulsion of feeder vessels may be catastrophic. In our case, CPC was not required to perform the ETV and therefore did not factor in the findings of postoperative VGAM thrombosis. However, given whether such procedural variation (i.e., CPC) contributes to ETV success in the short- or long-term remains yet to be established, we recommend significant consideration on the merits to choroid plexus cauterization in this subpopulation of ETV patients on a case-to-case basis.

Limitations to our study include those inherent to most case reports given the rarity of such pathology and inability to draw generalizations. The precise etiology behind spontaneous lesional thrombosis remains speculative. The findings herein merit further investigation in the form of multicenter prospective registries or international cohort studies to derive further meaningful data on the management of these complex cerebrovascular lesions.

## Learning points

- Hydrocephalus may present due to aqueductal obstruction, intraventricular hemorrhage, or venous hypertension leading to absorptive deficit in VOGM patients.
- Endoscopic third ventriculostomy is a safe alternative option to ventriculoperitoneal shunting for VOGM patients presenting with symptomatic hydrocephalus where endovascular embolization is no longer a feasible option.
- Further studies comparing endoscopic third ventriculostomy to ventriculoperitoneal shunting in VOGM patients are warranted to investigate complication rates and long-term outcomes
- In certain instances, endoscopic third ventriculostomy in VOGM patients may also induce spontaneous thrombosis of the cerebrovascular lesion in delayed fashion as reported herein

## Data Availability

The datasets presented in this article are not readily available because of ethical and privacy restrictions. Requests to access the datasets should be directed to the corresponding author.

## Supplementary material

SUPPLEMENTARY TABLE S1 Literature review of pediatric VGAM-related hydrocephalus treated by endoscopic third ventriculostomy (ETV).
